# Ingredients for success: Factors associated with successful integration of depression care into non-communicable disease clinics in Malawi: A mixed-methods analysis

**DOI:** 10.1371/journal.pmen.0000532

**Published:** 2026-01-06

**Authors:** Abigail M. Morrison, Chifundo Colleta Zimba, Melissa Stockton, Jullita Kenala Malava, MacDonald Mbota, Maureen Matewere, Harriet Akello, Christopher F. Akiba, Kelsey R. Landrum, Vivian Go, Mina C. Hosseinipour, Bradley N. Gaynes, Michael Udedi, Brian W. Pence

**Affiliations:** 1 Department of Health Behavior, Gillings School of Global Public Health, University of North Carolina at Chapel Hill, Chapel Hill, North Carolina, United States of America; 2 The University of North Carolina Project, Lilongwe, Malawi; 3 Department of Epidemiology, Gillings School of Global Public Health, University of North Carolina at Chapel Hill, Chapel Hill, North Carolina, United States of America; 4 Malawi Epidemiology and Intervention Research Unit (MEIRU), Karonga, Malawi; 5 RTI International, Research Triangle Park, Durham, North Carolina, United States of America; 6 Department of Medicine, School of Medicine, University of North Carolina at Chapel Hill, Chapel Hill, North Carolina, United States of America; 7 Department of Psychiatry, School of Medicine, University of North Carolina at Chapel Hill, Chapel Hill, North Carolina, United States of America; 8 Ministry of Health, Lilongwe, Malawi; Flinders University, AUSTRALIA

## Abstract

Depression is a leading cause of disability worldwide that disproportionately impacts low- and middle-income countries (LMICs). Evidence-based depression care options are often limited in LMICs and poorly integrated into existing healthcare systems. Improving such integration is crucial to improving patient outcomes and reducing disability. In Malawi, a cluster-randomized trial of implementation strategies to integrate depression care into non-communicable disease clinics was conducted at 10 healthcare facilities from 2019 to 2021. Some clinics were highly successful in integrating depression care while others were less successful in both arms. This post-hoc mixed-methods analysis (01/10/2023 and 31/05/2024) combined quantitative clinic performance indicators with qualitative endline key informant interview data to identify factors other than the tested implementation strategies that differentiated clinics that effectively integrated depression treatment from those that did not. Most participants reported several implementation barriers that were present across nearly all clinics. These pervasive barriers included access to resources, provider attitudes, and provider turnover. Differentiating factors, which when present allowed successful clinics to overcome pervasive barriers and when absent made the barriers difficult to overcome, included clinic coordinator engagement, management engagement, clinic ownership, and adequate training. Differentiating factors that facilitated implementation of depression care integration, even in the presence of commonly identified barriers, should be prioritized as targets for future implementation efforts.

## Introduction

Depression is a leading cause of disability worldwide with most of the burden in low- and middle-income countries (LMICs) [1,2]. Globally, 970.1 million people suffer from mental disorders with 279.6 million people experiencing depression [1]. Approximately 3440.1 per 100,000 people experience depression worldwide [1] while in sub-Saharan Africa 4540.4 per 100,000 people experiences depression [1].

Common evidence-based treatment options for depression include both pharmacotherapy (e.g., antidepressants) and psychotherapy (e.g., cognitive behavioral therapy) [3]. These treatment options are limited in LMICs. Implementing evidence-based depression treatment programs is essential to expanding access to care. Efforts to increase access to mental health care through task-shifting and integration of mental health services into primary care have been found to be effective methods to increase depression care in LMICs, but these methods have faced challenges of scale-up in the region [4,5]. Research on implementation strategies to support scale-up of depression care integration have been limited, and most studies have only been evaluated as part of pilot studies [6]. There have been few studies that have evaluated scale-up strategies in randomized trials [6].

In Malawi, access to mental health care is challenging in part because there are few mental health professionals and mental health services are not fully integrated into primary healthcare [7]. Barriers to implementing integrated depression care in Malawi include limited medical staff, medication shortages, and negative provider attitudes, amongst others [8–12]. While these barriers are challenging in low-resource settings, some clinics succeed in the face of common barriers while other clinics cannot effectively integrate care. We applied a post-hoc mixed-methods approach, after the conclusion of a 10-site clinic-randomized trial of strategies to support depression treatment integration into non-communicable diseases (NCD) care, to explore factors other than the intervention package that differentiated clinics that successfully integrated depression treatment into general medical care from clinics that did not.

## Methods

The primary objective of this post-hoc analysis of a completed implementation science trial was to identify factors other than the tested implementation strategies that distinguished clinics that were able to effectively integrate depression treatment despite barriers from clinics that were not able to overcome the same barriers to integration. We used a mixed-methods approach with data from a cluster-randomized trial of strategies to support integration of depression treatment into NCD care. In this analysis, we combined quantitative clinic performance indicators with qualitative information from endline key informant interviews to triangulate reasons for successful integration and provide a rich description of how clinics were integrating depression treatment.

### Parent trial

This analysis uses data from a cluster-randomized implementation trial, the Sub-Saharan Africa Regional Partnership (SHARP) for Mental Health Capacity Building trial [13]. The trial aimed to facilitate ongoing Malawi Ministry of Health efforts to scale up an evidence-based depression treatment program within noncommunicable disease (NCD) departments at 10 of the 29 secondary-level district hospitals in Malawi representing all three regions and covering roughly one-third of the population [14]. The study sites were purposefully selected to represent both rural and urban populations as well as geographic regions across Malawi. The Ministry of Health prioritized integration of mental health care into the standard care provided at NCD clinics due to the high prevalence of depression in this population [13]. Additionally, NCD clinics were believed to be well-suited to integrating mental health care as they were already designed to provide ongoing care for chronic conditions and had the means to follow patients over time. Data collection was facilitated by research assistants who were paid by external partners. Data management and analysis was conducted by both local and external partners.

### Depression screening and treatment program

At each study site, training was provided to three clinicians who in turn trained their peers. Training was facilitated by both external and local partner organizations. Clinicians were trained to screen and monitor patients for depressive symptoms with the Patient Health Questionaire-9 (PHQ-9) and provide appropriate treatment using the Friendship Bench problem-solving therapy model and/or measurement-based antidepressant prescription [13,15–17]. Patients identified with mild depression (scores 5–9) were referred for Friendship Bench; a problem-solving therapy delivered by trained expert peers in which patients are guided to identify problems and work towards solutions over the course of six sessions [13,14]. Friendship Bench was originally developed in Zimbabwe, but has been adapted for use in Malawi [15,18]. Patients identified with moderate to severe depression (scores ≥ 10) were prescribed antidepressants (fluoxetine or amitriptyline). Clinicians were also trained to respond to suicidal thoughts and follow-up on depression treatment. Patient follow-up included monthly reassessments of depression symptoms for at least three months. The dose of the prescribed antidepressant was increased if PHQ-9 scores were still ≥ 10. Antidepressants were obtained by the clinics through the national medical plan. Detailed descriptions of the depression screening and treatment program have been previously published [13,14].

### Implementation strategies

The parent study compared two implementation strategies aimed at supporting the Ministry of Health’s efforts to integrate depression care into NCD clinics [13]. The basic implementation strategy consisted of training and supporting a facility provider as a program champion to spearhead the integration process, including training and supporting their colleagues. Again, training was facilitated by both external and local partner organizations. The champions were responsible for training providers to provide depression care, maintain patient records to track screening and treatment efforts, supervise the Friendship Bench Counselors, supervise other clinicians, maintain a supply of antidepressant medications, and report monthly to the Malawi Ministry of Health [13]. The enhanced implementation strategy consisted of the basic training, plus quarterly external supervision and mentorship visits from clinical experts and Ministry of Health staff. The external supervision visits included a discussion of challenges, observation of clinic services, an audit of paperwork, review of integration efforts, and feedback and recommendations to the champion and other clinic leadership. The parent study examined whether the enhanced implementation strategy resulted in higher quality mental health care and better patient outcomes [13]. The trial ran from 09/05/2019 until 31/12/2021.

### Sites and patient participants

Ten hospitals spread throughout the northern, central and southern regions of Malawi participated in the study comparing implementation strategies. Nine of the District Hospitals served rural/semi-urban catchment areas of the Central Region, and one hospital served patients from an urban area. Medical services at each of these facilities are directed by a District Medical Officer (DMO); two hospitals share a District Medical Officer. Clinics were eligible for inclusion as a study site if they were the district’s primary source of hypertension and diabetes care and provided care to at least 80 patients seeking care for hypertension and diabetes per month. Sites were randomly assigned to either the basic or enhanced implementation strategy arm with constraints to ensure balance between arms [13,14]. All patients presenting to the NCD clinic were eligible for depression screening and treatment. Patients who presented for care at these clinics were not responsible for the cost of their visit or any medications prescribed as Malawi provides an Essential Health Package that defines which healthcare services are provided free of charge at public health facilities. These services defined in the Essential Health Package include depression screening, treatment, and antidepressants.

### Parent trial primary results

Across the two study arms, a total of 60,774 visits were eligible for data collection (i.e., visits for patients between 18 and 65 years and presenting for hypertension and diabetes) and a total of 589 patients were enrolled to provide patient outcome data. Primary outcomes (fidelity to depression screening, treatment initiation, and follow-up protocols) and secondary outcome (depression-related patient outcomes) results have been previously reported [14]. In brief, the study arms did not differ significantly on many fidelity indicators including rates of initial screening for depression (58% in the enhanced strategy arm compared to 53% in the basic strategy arm) or appropriate treatment initiation (98% in the enhanced strategy arm compared to 99% in the basic strategy arm). Follow-up fidelity for antidepressants was significantly higher in the enhanced strategy arm (90%) compared to the basic strategy arm (22%) and for Friendship Bench Counselling (83% compared to 15% respectively). Depression remission at 3 months after starting treatment was higher in the enhanced strategy arm (55%) compared to the basic strategy arm (36%), an advantage which remained true at 6 and 12 months [14].

### Rationale for current analysis

Based on the findings from the parent study, additional questions were raised regarding why some clinics were able to more successfully integrate depression care into NCD clinics (i.e., higher fidelity to depression care protocols) while others were not, *regardless* of the study arm and thus, the implementation support they received. Patient health outcomes were not considered in this analysis as the focus was on the process of integration. While some fidelity measures differed between implementation strategy, the initial screening for depression and treatment initiation did not. This current analysis was conducted to identify factors impacting successful depression care integration that existed outside of the implementation intervention strategies.

### Data analysis

Convergent mixed methods [19] were used to compare clinic performance with regards to implementation and fidelity of depression screening and treatment initiation as well as treatment delivery over time. Data for this analysis was accessed between 01/10/2024 and 31/05/2024.

### Quantitative data

Quantitative data were collected from 01/10/2019 to 30/11/2021 [14]. For this current analysis, five performance indicators from each clinic were examined for use in an overall summary score. Performance indicators were chosen if they related to clinic performance and had sufficient variability between clinics to help differentiate higher and lower performing clinics. Clinics were not stratified by study arm because results from the parent study indicated that the basic and enhanced implementation strategy had similar effects on depression care integration fidelity and that there must be other factors influencing fidelity and integration efforts.

Four performance indicators were from the quantitative data:

- Screening fidelity: of screening-eligible visits, the percentage where screening was completed (screening %);
- Treatment initiation fidelity: of medication treatment-eligible visits, the percentage where medication was initiated (treatment medication initiation %);
- Follow-up Fidelity: of follow-up visits in the first 3 months after initiating depression treatment, the percentage where depression treatment was appropriately continued (follow-up fidelity %); and
- Suicide protocol fidelity: of visits in which suicidal thoughts were indicated, the percentage where a safety assessment was completed (suicide risk assessment %).

At the conclusion of the trial, the three study coordinators with experience working with all the facilities for the duration of the trial assigned a consensus summative rating to each facility as being highest performing, high performing, inconsistent, low performing, or lowest performing to make the fifth and final indicator. These ratings were assigned prior to reviewing the quantitative indicators listed above. For this analysis, the levels in this ordinal scale were assigned numerical values ranging from 55 (lowest performing) to 95 (highest performing) such that the resulting variable had approximately the same mean and standard deviation as the clinics’ average performance score on the other four indicators.

Each clinic was assigned a final performance score calculated as the weighted average of the five indicators. All indicators received the same weight apart from follow-up fidelity, which both had a very large range and was based on a small denominator (<10 eligible visits) in several clinics and was therefore judged to be not as reliable as the other indicators; this indicator was given only half the weight of the others in calculating the overall final performance score. The three clinics with the highest scores were considered to be “high performing” clinics while the three clinics with the lowest scores were considered to be “lower performing”. This helped to categorize the clinics into roughly equal groups.

### Endline qualitative research

Trained interviewers conducted endline qualitative interviews from November 2021 to January 2022 by phone (n = 34) or in-person in a private place at the study sites (n = 5) to 1) determine how workload, provider turnover, and the involvement of external partner organizations impacted the integration of depression care into NCD services; 2) understand the impact of leadership and implementation climate on overcoming integration barriers; and 3) understand how some sites were able to overcome barriers and achieve strong integration while other sites were not. The interviews lasted approximately 1 hour and were conducted mostly in English with occasional sentences in the local language, Chichewa. The interviews were recorded, transcribed verbatim, and translated as necessary.

Thirty-nine purposively selected key informants, individuals who could provide unique insight into the integration process, were included to represent a diverse array of experiences across the integration team that would provide a more comprehensive picture of the integration process. Key informants included the District Medical Officer (DMO), the NCD Clinic Coordinator, an NCD provider, and the SHARP Research Assistant from each of the ten healthcare facilities (note, two facilities share a District Medical Officer). The District Medical Officers were physicians by training and led the clinical teams at the district hospitals. They have the authority to address NCD clinic-level implementation problems (e.g., staff shortages). The NCD coordinators were either physicians, physician assistants/clinical officers, or nurses who worked directly with NCD patients and were responsible for the coordination of NCD care at the designated NCD department [12]. NCD providers included nurses and clinicians who were involved in screening patients and either referring patients for Friendship Bench therapy or prescribing antidepressants. The District Medical Officers, NCD coordinators, and NCD providers were all MOH employees. Research Assistants (RAs) employed by the study consented and surveyed patient participants at the study sites and abstracted clinical data. RAs were not directly involved in patient care nor directly facilitating program implementation.

The codebook was developed iteratively using both inductive and deductive strategies. Parent codes were chosen based on the interview guides and study aims. Child codes were developed as key themes emerged. The transcripts were coded and organized in NVivo 12 by seven team members [20]. Each team member initially coded the same two transcripts. They then met to review the coding line-by-line to ensure inter-coder reliability (ICR) and make adjustments to the codebook and coding strategy as necessary. Thereafter, the team then met weekly to discuss the coding and analysis progress and adjudicate any coding discrepancies. Upon completion of coding, the team developed matrices to conduct content analysis. These matrices helped organize the data, clarify key themes, and facilitate comparisons across facilities and key informants [21]. The matrices were then used to create memos (written descriptions and explanations of key themes and study aims with representative quotes for each theme) [22]. One memo focused on the key implementation barriers and facilitators and how these impacted clinic implementation of depression screening and treatment activities. The research staff members involved in writing that memo also ranked clinics from lowest performance to highest performance based on their in-person observations and experiences and their overall impressions from reading the interview transcripts. As mentioned above, this ranking was used in the calculation of each clinic’s overall performance score.

Using the barriers and facilitators identified in the memo and matrices, a second round of coding was completed by AM to systematically identify mentions of these reported implementation barriers and facilitators. AM then created additional matrices to indicate the presence, absence, or combination of implementation barriers or facilitators at each clinic [21]. These matrices were compared against the final clinic performance scores (from the quantitative data analysis) to determine if there were any patterns that might explain how lower and higher performing clinics differed [23]. For the interview with the District Medical Officer from Karonga and Chilumba, barriers and facilitators were noted only when they referred to a specific site.

### Ethical considerations

The University of North Carolina Biomedical Institutional Review Board and the Malawian National Health Sciences Research Committee approved this study (#21709 and #21802). All study participants provided written informed consent. Authors had access to information that could identify individual participants during and after data collection. Additional information regarding the ethical, cultural, and scientific considerations specific to inclusivity in global research is included in the Supporting Information (S1 Checklist)”.

## Results

### High and low performing clinics

The overall performance scores and clinic performance rankings can be found in Table 1. There were 3 high performing clinics, 4 medium performing clinics, and 3 low performing clinics. The quantitative indicators aligned well with the research staff rankings. Performance scores reflect the findings from the parent study that some clinics were able to successfully integrate depression care (high performing) while others were not (low performing) regardless of implementation intervention strategy.

**Table 1:** Clinic performance by implementation and fidelity measures.

	Strategy (Basic/ Enhanced)	Screening %	Treatment Medication Initiation %	Follow-up Fidelity %	Suicide risk assessment %	Research Staff Ranking	Overall performance score	Final Rank
High performing clinics	E	99.6	84	33.3	97.5	Highest	87.3	1
	B	96.6	90.9	32.1	98.5	High	86.0	2
	E	68.3	93.4	68.2	89.7	High	82.3	3
Medium performing clinics	B	57.5	95.2	38.1	97.8	Medium	76.6	4
	B	62.1	92.1	14.9	91.4	High	75.1	5
	E	68.6	63.3	90.5	89	Low	73.6	6
	E	24.7	100	54.3	99.4	Medium	72.5	7
Low performing clinics	B	55.9	87.1	11.4	87.6	Low	67.0	8
	E	45.7	65.2	82.4	85.7	Lowest	65.1	9
	B	14.5	82.4	25	93	Medium	61.6	10

### Pervasive implementation barriers

In qualitative interviews, three factors were regularly reported and identified as barriers to implementation of and fidelity to the depression screening and treatment protocol at all or nearly all facilities. These factors included access to resources, provider attitudes towards depression screening and treatment integration, and frequency of provider turnover.

#### Access to resources.

Access to resources, specifically a shortage of medication, providers, and clinic space, was the most often reported barrier and was reported by all ten sites. This barrier was not correlated with clinic performance. For instance, one clinic coordinator at a lower performing clinic reported that:

*“…sometimes it happens that the clinicians have to multitask. You will find that maybe a clinician is supposed to work in NCD and at the same time he has to work in OPD. In this case you find that there are 3 to 4 clinicians or even 2 and it becomes a difficult task for them. As a result they just pose general questions to the clients and if the answer is no, they don’t proceed with the other questions as a result they miss out clients who are depressed.” (Clinic Coordinator 1, low performing clinic)*

While a District Medical Officer at a higher performing clinic reported that,

*“There is also a shortage of drugs if you are screening those that have got major depression lately we have been having challenges to make sure that we have got constant drugs that we need to have” (District Medical Officer 1, high performing clinic)*

Shortages of providers impacted care because providers had high patient volumes to attend to and limited time with each patient. Adding depression screening to an already short appointment increased the workload and amount of paperwork required to be completed by providers. This made screening difficult and burdensome to providers. Additionally, medication shortages left patients who were prescribed antidepressants unable to fulfill their prescriptions. This was discouraging to both patients and providers.

#### Provider attitudes.

Another barrier reported by nine of the ten clinics was providers having negative attitudes towards integrating depression screening and treatment into the NCD clinic responsibilities. This barrier was present at both high and low performing clinics. Negative attitudes were described as providers not wanting to screen for depression, not believing depression was an important health concern, feeling like screening was a waste of time, not screening unless they were being supervised, or not wanting to work in the NCD clinic at all. For example, one clinic coordinator at a lower performing clinic reported that:

*“even [providers] who were trained, they don’t administer the questionnaires because they say it is tiresome” (Clinic Coordinator 2, low performing clinic)*

While a research assistant at a higher performing clinic specifically noted how attitudes impacted depression care integration:

*“[it] is just to do with the attitude that they have, some people naturally do not like the work…It all depends on the willingness of the person who has come to NCD on that particular day. If he does not have passion, or he does not want to screen, he is not going to screen…[the providers] complain that it is just an addition to the work that they do, or that it is an unnecessary baggage that was added to their load” (Research Assistant 1, high performing clinic)*

Provider attitudes were impacted by the increase in workload and feelings of not having enough time with each patient. Attitudes were also impacted by how important of a health condition the provider thought depression was. Providers who did not feel depression was a major concern were less inclined to screen and often complained about the added work.

#### Provider turnover.

A third barrier reported by nine of the ten clinics was frequent provider turnover, especially regarding providers trained in depression screening and care. Provider turnover was reported as a barrier at both high and low performing clinics. Provider turnover occurred when providers moved to other clinics within the same health facility or another health facilities or went back to school. When providers left, it exacerbated the existing shortages of trained providers at the NCD clinics. One research assistant at a lower performing clinic reported on the scope of the problem:

*“looking at the numbers it is about 50 to 75% who have moved…There is an effect because you find that not every patient is screened for depression and also the numbers that are picked for depression are too low because some are not screened for it. So it might mean that someone might not be attended to the screening for depression.” (Research Assistant 2, low performing clinic)*

As this key informant also noted, the provider shortage meant that some patients who were likely depressed were not identified through screening and thus did not have care initiated. Provider turnover was also an issue at higher performing clinics. Another research assistant at a higher performing clinic noted that:

*“Ah…the impact has been huge because with them leaving, there have to be others; new ones in the clinic and these are the providers who have not gotten any training and for them to grasp what is really required of them, it takes time. So when you have a new clinician, it takes about two weeks for them to be fully oriented to be able to do the work. So I think that has been one of the challenges that has been taking us back during the integration.” (Research Assistant 3, low performing clinic)*

This key informant comments on the gaps in institutional knowledge when trained providers leave. The remaining providers have not necessarily been trained. There is then a lag in depression management while the new providers receive training.

### Differentiating factors of implementation

Four factors differentiated those clinics that did vs. did not successfully implement integrated services, independent of the presence of universal barriers noted above: support and engagement by clinic coordinators, support and engagement by District Medical Officers, local ownership of depression screening and treatment activities by the clinic, providers, and management, and adequate training of the providers to implement depression screening and care. Key informants at lower performing clinics regularly reported the absence of these factors to be barriers that exacerbated aforementioned implementation barriers while high performing clinics reported the presence of these factors as facilitators that allowed the sites to overcome other existing implementation barriers.

#### Clinic coordinator engagement and support—Lower performing clinics.

Lower performing clinics often reported that clinic coordinators were not supportive or engaged in depression care integration. Lack of support and engagement was described as clinic coordinators not being present at the clinic, not believing depression care is important to patient health, having negative attitudes, not encouraging other providers to screen, or not being effective leaders (e.g., the other providers did not respect their authority). One research assistant at a lower performing clinic reported that the clinic coordinator’s negative attitude:

*“…really affects because s/he is the one who is supposed to be in the forefront but if s/he is not active, there is no way the other clinicians can promote the services and you find that depressed patients are not being screened and they are denied access to care” (Research Assistant 4, low performing clinic).*

This clinic coordinator’s negative attitude discouraged other providers from screening and signaled that depression was not an important health concern. It also gave permission to other providers to not screen or view depression care as a negotiable part of NCD care. The coordinator did not lead by example and screening suffered as a result. Another research assistant at a lower performing clinic described how a clinic coordinator was not able to lead saying:

*“the coordinator seems to have no power to bring some providers to be at the NCD clinic…So that also gave a tough problem to the coordinators, so sometimes you could see as if they have powers but sometimes also they could be seen as if they don’t have much powers to organize the clinic” (Research Assistant 5, low performing clinic)*

This key informant describes how the clinic coordinator had trouble getting other providers to work at the NCD clinic despite them being assigned to duty. Even though the coordinator had the responsibility and authority to create the roster of on-duty providers at the NCD, the providers did not necessarily respect this or come to work.

#### Clinic coordinator engagement and support—Higher performing clinics.

While lower performing clinics struggled with clinic coordinator engagement and leadership, high performing clinics often reported that clinic coordinators were supportive and highly engaged in depression screening and treatment activities. This was described as clinic coordinators having an interest in depression care, having a positive attitude, encouraging other providers to screen, and actively supervising other providers to ensure they were screening patients.

One research assistant at a higher performing clinic reported:

*“To be honest, the Coordinator we had here has been very active. His attitude towards this integration has been very positive on this depression integration…It had an impact because he has been making sure that all patients are screened for depression and that there is care that the patients required. If there are patients who are positive for depression they should be treated accordingly. He was also reminding other clinicians to screen for depression.” (Research Assistant 3, high performing clinic))*

Here, coordinator engagement was a facilitator for depression care integration rather than a barrier as it was lower performing clinics.

#### Management personnel engagement and support—Lower performing clinics.

Similarly, at lower performing clinics, a lack of support and engagement by management personnel was also a barrier while supportive and engaged management was a facilitator at higher performing clinics. Support and engagement by management mostly referred to involvement of the District Medical Officer and their presence and help at the clinic, their attitude towards depression care integration, and their awareness of and responses to challenges the NCD clinics were facing. One research assistant at a lower performing clinic noted that the District Medical Officer, while aware of the challenges at the clinic, did not address them:

*“I think it affects the depression care negatively. That is because if he is aware of the problems that are being faced at the NCD clinic and nothing is being done, I think the clinicians also will not feel motivated because they will think that the DMO is aware and is doing nothing, they will also just stand back and watch. I think they feel like they will not be… they think that the blame will not be on them because if the DMO is aware, the DMO would be answerable. So, I think the clinicians also sit back thinking that the DMO knows and if he is doing nothing, they can also do nothing” (Research Assistant 6, low performing clinic)*

#### Management personnel engagement and support—Higher performing clinics.

In contrast, higher performing clinics reported that management personnel were supportive and engaged in depression care integration. One clinic coordinator at a higher performing clinic said of their relationship with their District Medical Officer:

*“It has been a good relationship I would say, because he was very involved in all the activities. He showed interest in previous activities we had, yes, so we talk, we really talked about the progress itself, how it has been, where we are at all time. Yah, so it has been a good relationship. We could go there and then you’d see this reception whereby he’s very interested to know what is happening…In level of support I’d say since he’s there to support us I’d say that it’s not that difficult for us like to involve other clinicians because there is someone there at the top who is really supporting us and who is understanding what is happening to them. So him being there to supporting us at the clinic, this has given us morale to push on like to... despite the challenges that we have. It gives us that strength” (Clinic Coordinator 3, high performing clinic)*

At this clinic, the providers have been encouraged by the District Medical Officer to screen for depression. Management has also helped motivate providers by having a positive attitude and being involved in the activities at the clinic. This support helped the clinic and providers “push on” through other challenges to depression care integration.

#### Clinic ownership—Lower performing clinics.

Lack of clinic ownership of the depression screening and treatment program was a barrier in lower performing clinics while clinic ownership was a facilitator in higher performing clinics. Lack of ownership was described by key informants as providers or management seeing depression screening and treatment activities as part of a research study owned by the research team and not part of the regular clinic duties determined by the Ministry of Health. This was evidenced by depression and screening activities only taking place when the research assistant was present, or key informants stating that providers would not continue providing these services once the study was complete. One research assistant at a lower performing clinic reported that the research team’s presence at the clinic:

*“...has affected both positively and negatively because when UNC [the University of North Carolina] comes things work perfectly well and even when the supervisors come, things work well. However, it has also affected negatively because when those people leave, they stop because they think the owners have gone.” (Research Assistant 4, low performing clinic)*

At this clinic, providers viewed depression screening and care as solely a research study activity and not a routine duty of the clinic as care for other health issues like diabetes and hypertension were. As such, providers did not screen every patient who presented to the clinic; they only screened when they felt the research study was taking place and data needed to be collected.

#### Clinic ownership—Higher performing clinics.

In comparison, clinics that had stronger ownership of depression care often reported having plans in place to sustain depression care after the study ended, viewed depression care as core duty of the clinic, one that was as integral to patient health, and a program that was ultimately owned by the Ministry of Health. These higher performing clinics would have providers regularly screening patients even without supervision from the research team. One District Medical Officer at a higher performing clinic said of depression care:

*“I would say ours is already into the routine activities despite the study having reached its targets, we continue to screen because of the positive outcome that we have experienced through the study. So the plans are already there and as I said, some of the activities will be included in the [District Implementation Plan] so that we make sure that the depression screening should continue” (District Medical Officer 2, high performing clinic)*

At this clinic, management was already making plans to include depression screening and care in their annual planning to formalize it as a routine clinic duty. Providers were already treating it as a core activity because they were seeing the benefits of screening, but management also wanted to ensure that there was funding for sustained care. The clinic coordinator at this clinic also said:

*“Orientation of staff either nurses or clinicians...everyone that is coming to a clinic they have to understand that there is this thing that we expect on at the clinic” (Clinic Coordinator 3, high performing clinic)*

Despite reporting provider turnover and the loss of trained providers, the coordinator was determined to make sure new providers understood that depression care was integral to clinic activities and that they would be expected to screen all patients. Setting this expectation demonstrated that the clinic had embraced depression screening and considered it a non-negotiable service it provided to patients.

#### Adequate training—Lower performing clinics.

Inadequate training was a barrier in lower performing clinics, while adequate training was a facilitator in higher performing clinics. Most NCD clinics did not have dedicated or permanent staff, but instead had staff rotating through the different departments at the hospital. In light of high facility turnover and departmental rotations, the NCD clinics could be staffed by providers that had not attended the initial depression screening and treatment training. As such, adequate provider training specifically refers to the need for new staff to be trained on the depression management protocol. Low performing clinics often reported that no training was done or that training was informal, on-the-job training (e.g., being trained while one provided care for patients), or mentorship by a more experienced provider. A provider at a lower performing clinic discussed how the District Medical Officer addressed provider shortages:

*“…the DMO sometimes ensures that the clinic is covered by bringing in clinicians maybe the interns who are not well trained just to cover the clinic. So maybe these interns were just briefed for a short time maybe they were trained by the Coordinator but their knowledge is not up to the optimal care. So that does affect depression care at the clinic.” (Provider 1, low performing clinic)*

New providers did not have enough training to provide a high level of care to patients. Further, providers who did not receive formal training often had negative attitudes towards depression care integration. One research assistant at the same clinic reported:

*“I feel like the providers who started this welcomes it very much maybe because it was new to them. The training had an impact because they mastered it as opposed to the new providers who don’t have adequate knowledge because they are not specifically trained but they are just oriented by their fellow providers. So they seem not to have much interest as compared to the old providers.” (Research Assistant 4, low performing clinic)*

#### Adequate training—Higher performing clinics.

Higher performing clinics were more likely to report adequate training of providers. Adequate training for these providers was described as the provision of refresher training or some other more formal training opportunity. One research assistant at a higher performing clinic reported that the clinic coped with provider turnover by providing training:

*“Mostly when a provider left, they were doing a training and it was very easy for the new providers to catch up…I think if they can continue doing meetings, on-job trainings, refresher trainings…” (Research Assistant 7, high performing clinic)*

At this clinic, the on-the-job training and the more formal refresher training helped mitigate the effects of provider turnover and made it easy for new providers to integrate depression care. Adequate training at this clinic helped also improve provider attitude. When asked what factors contributed to providers enjoying the screening for depression, one District Medical Officer said:

*“I think one of the things is applying the knowledge that they gained through the trainings that we had and of course the positive outcomes from the depression screening that we were providing […] we have seen that some of the [other NCD conditions] have improved having addressed the depression issues […] And because we have been able to screen the patients and have the outcome, that I think makes [the providers] to be vigilant with the exercise […] when you screen and you find that one is depressed and you help them out of that situation, that is also… that is why we are positive about it and we would want this to continue” (District Medical Officer 2, high performing clinic)*

Being able to recognize the importance of screening for depression and see the positive outcomes for patients was motivating to providers.

## Discussion

This mixed-methods study of factors influencing the success of depression treatment integration into noncommunicable disease care in ten health facilities in Malawi, identified both pervasive barriers and differentiating factors between clinics with successful and less successful implementation success. Pervasive barriers that were present in all or nearly all clinics, regardless of implementation success, included access to resources, provider attitudes, and provider turnover. Differentiating factors, that when present appeared to allow some clinics to overcome these pervasive barriers and when absent appeared to make the barriers difficult or impossible to overcome, included clinic coordinator engagement, management engagement, clinic ownership, and adequate training. Differentiating factors that facilitated implementation should be prioritized as targets for future implementation efforts.

### Pervasive barriers to depression care integration

The pervasive barriers provide insight into the barriers that are present in clinics across Malawi and other LMIC healthcare systems. Prior literature has reported on the deficit of healthcare workers [24] and essential medications [25] in Malawi, so it is not surprising that access to resources (including medications) and provider turnover were reported to be barriers at most clinics. Previous qualitative work in Malawi on patient and provider attitudes around depression have indicated that depression is not always viewed as a clinical disorder or a disease with a biological origin [26]. This may contribute to the findings in this study that providers often report negative attitudes towards integrating depression screening and management into NCD clinics and do not always feel that depression care was a priority for clinical care.

### Differentiating factors to depression care integration

While these pervasive barriers were identified at all or nearly all sites and hindered implementation efforts generally, participants also identified differentiating factors that, when present, allowed some sites to succeed in spite of these pervasive barriers. These factors helped explain why some clinics successfully integrated depression care while others did not. The presence of these factors as facilitators allowed high performing clinics to overcome the pervasive barriers and integrate depression care. The absence of these factors at lower performing clinics only added to the number of barriers the clinics faced and therefore hindered integration. Each of the differentiating factors are good candidates for future implementation strategies because the presence of these factors as facilitators allowed clinics to overcome other barriers and provide higher quality depression screening and care.

For example, negative provider attitudes could be addressed by each of the deterministic factors. First, having a clinic coordinator who was highly engaged and actively supervising the providers helped ensure patients were screened regardless of providers having negative attitudes towards screening. Engaged leaders also helped signal to providers that depression care was important and improves provider attitudes in spite of other challenges. Having engaged leaders and management personnel is key to integrating mental health services into primary healthcare [11,27,28]. Methods to improve leader engagement should be evaluated in future implementation studies. Second, ownership over depression care also helped address negative provider attitudes because depression care was seen as a core clinic responsibility and not something that each provider could decide to conduct or not. In Uganda many primary care providers do not see mental health care as a priority and believe that their only role is to refer patients to more specialized care [27]. When mental health care is not owned by the providers, care is not well integrated into existing services. Further, negative provider attitudes were also impacted by having adequate training. Training was helpful in educating providers about the importance of depression care and improving provider attitudes towards screening. Similarly, in South Africa and Zambia, primary care workers who had more formal education were better able to identify mental health disorders among patients [29]. As such, training was a critical factor for successfully integrating mental health care into primary health care services [29]. In Zambia there has been a call for increased training to address stigmatizing attitudes towards mental health disorders and patients with mental health disorders [30]. Training may only be possible in conjunction with committed leadership and increased funding [30]. In Uganda, providers with only basic mental health training did not see mental health care as a primary responsibility (also relating to lack of ownership) and had negative attitudes towards mental health care [27]. Even providers who had been oriented to mental health disorders had low interest in providing mental health care [27]. More formal and targeted training is necessary to improve provide attitudes towards mental health care.

Clinics were also able to successfully address high provider turnover when they also had adequate training. All new providers needed to be trained on depression screening and management. Without ongoing training, especially formal training, provider turnover led to knowledge gaps and a decrease in depression care. Formal training improves mental health literacy and the ability of providers to identify mental health disorders among their patients [29].

Lastly, lack of access to resources could be addressed indirectly by strong clinic coordinator or District Medical Officer engagement. Engaged leaders were more aware of the problems with access to resources and could better advocate for increasing staffing or improving supply chain management. Often when leadership does not prioritize mental health or view mental health care as a duty, mental health services only receive limited health resources [27]. Engaged leadership and an increase in funding for mental health education is essential to improve mental health care [30]. Second, adequate training helped to address some of the provider shortages in clinics. Shortage of providers is a barrier across Malawi, and providers trained in depression care are especially rare. This is also the case in Uganda where the lack of trained providers was identified as a barrier to integrating mental health care into primary care clinics [27]. In Zambia, increasing access to funding was necessary to increase training for providers, address negative provider attitudes, and improve mental health care [30].

### Limitations

There are a few limitations to this mixed-methods analysis. First, specific implementation factors may have been mentioned because the interview guide specifically asked about them. For example, the interview guides asked key informants to reflect on District Medical Officer engagement and also asked key informants to describe provider turnover and how turnover impacted depression care. This guidance may have led to key informants discussing barriers that they would not have otherwise noted. However, the interview guide arose out of the research team’s experience working with each of the sites and reflected the emphasis areas the research team thought would be relevant to the research question. Further, the guide was developed iteratively and adapted after the first few interviews to better address what key informants were discussing. Findings may have been impacted by social desirability. Some key informants may have spoken more positively about depression care integration and the facilitators because they wanted the research team to think favorably of their clinic. Regardless of this potential bias, key informants readily discussed implementation challenges, and their responses were aligned with clinic performance quantitative data. It should also be noted that while there were challenges to implementing this model of integration (depression care in NCD clinics), this option was chosen as it aligned with the priorities of the Malawian Ministry of Health focusing on integration of mental health care into other clinical services. There may be other pathways to expand access to depression care such as clinics specific to mental health. Another possible pathway to bolster screening efforts and better identify patients with depression could include machine learning approaches [31]. While medical personnel would need to be trained to use such methods and additional resources (*i.e.,* computers) would need to be available, machine learning approaches may more effectively identify patients with depression than traditional screening methods. Such methods warrant additional research. Lastly, this implementation study was disrupted by the SARS-CoV2 pandemic. However, the lessons learned from this study may be useful to future implementation efforts as health systems in low-resource settings continue to face a variety of expected and unexpected stressors.

## Conclusion

Clinics consistently identified three barriers to integrating depression care in Malawian NCD clinics: access to resources, provider turnover, and negative provider attitudes. Despite these pervasive barriers, some clinics were able to integrate high quality depression care into NCD clinics because they had adequate training for providers, engaged leadership, and ownership over activities related to depression care, while clinics that lacked these differentiating factors were not successful in overcoming the same barriers. We recommend that future research evaluate implementation strategies to integrate depression care into NCD clinics target 1) increased mental health care training for providers; 2) effective engagement of leadership; and 3) the promotion of clinic level ownership over integration efforts.

## Supporting information

S1 Checklist Inclusivity in global research. (DOCX)
